# An Interesting Civilian Case of Complex Maxillofacial Trauma Due to Target Fragmentation Following Bullet Impact and Review of the Branches of the Maxillary Artery

**DOI:** 10.7759/cureus.10484

**Published:** 2020-09-16

**Authors:** Brian Patterson, Sophia Sangar, Raja Gnanadev, George Makkar, Michael Neeki

**Affiliations:** 1 Surgery, Arrowhead Regional Medical Center, Colton, USA; 2 Head and Neck Surgery, David Geffen School of Medicine at University of California-Los Angeles, Los Angeles, USA; 3 Vascular Surgery, Arrowhead Regional Medical Center, Colton, USA; 4 Emergency Medicine, Arrowhead Regional Medical Center, Colton, USA

**Keywords:** trauma, external carotid artery, maxillary artery, maxillofacial, shrapnel

## Abstract

Serious morbidity and mortality for the operator and bystanders are associated with a lack of knowledge and failure to utilize appropriately manufactured targets. The management of firearm-related facial trauma is challenging and requires rapid intervention from a multidisciplinary team. We present a case of penetrating facial trauma secondary to the fragmentation of a homemade target. We highlight how firearm operators can optimize safety by matching ballistics with target selection and review pertinent vascular structures, including the terminal branches of the external carotid artery and branches of the maxillary artery. This case demonstrates that trauma physicians must be well-versed with complex maxillofacial anatomy and multimodal approaches to hemostasis.

## Introduction

Shrapnel-related maxillofacial injuries are well-documented, with clear guidelines in the military literature, but the incidence and reporting of such injuries in the civilian setting is uncommon [1-4]. Military experiences in the modern wars in Iraq and Afghanistan have contributed to clear guidelines for high-velocity and high-energy gunshot wounds to the face [1]. As with any trauma, following the Advanced Trauma and Life Support protocols is critical to the management of such injuries, as is the early integration of a multi-specialty team, including emergency department providers, critical care surgeons, vascular surgeons, and maxillofacial surgeons [1-2].

Given regional structures, shrapnel-related maxillofacial trauma often leads to complex injury, including bony, muscular, nervous system, and vascular injury [5]. We report a rare case of civilian shrapnel-related complex maxillofacial trauma due to target fragmentation following bullet impact. This report reviews the relevant anatomy with a focus on regional vascular structures, including the terminal branches of the external carotid artery and branches of the maxillary artery.

A review of the literature was conducted through Google Scholar, PubMed, and Google Books using keywords including, but not limited to, “maxillofacial shrapnel injury,” “civilian shrapnel injury,” “ballistic facial injury,” and “maxillary artery trauma.”

## Case presentation

**Figure 1:**
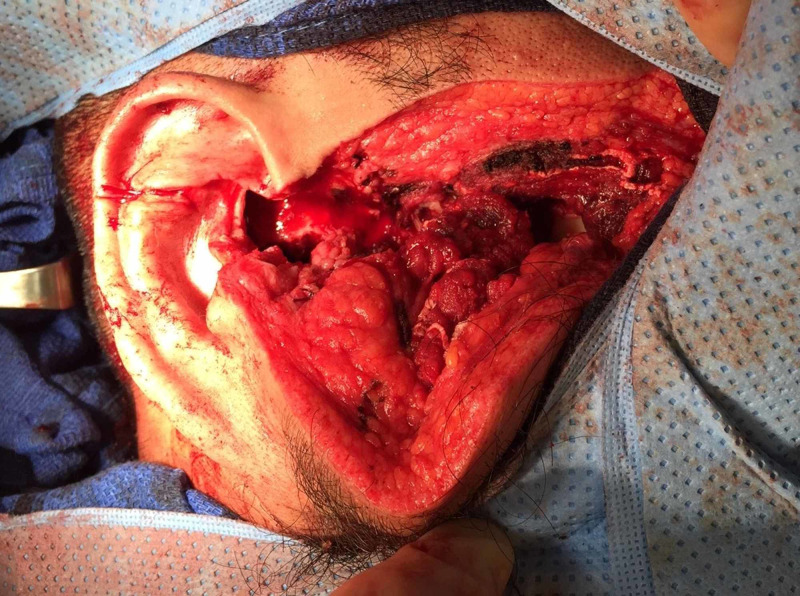
Significant right facial laceration in the maxillary and parotid regions

A 36-year-old male was brought to the emergency department (ED) with a deep facial laceration (Figure 1) after being struck by a target fragment from a homemade target after bullet impact. The patient was firing a 9-mm semi-automatic handgun. Per the patient, the target had been used frequently in the past and was constructed from wood and metal. After discharging his firearm multiple times, the patient reported that everything suddenly went black and he felt his body go limp. Emergency medical services reported 1 liter of blood loss en route.

Clinical findings

On exam, an 11-cm-deep, right-sided facial laceration extending from the lateral aspect of the right lip to the posterior right ear was noted. Further inspection revealed a full thickness laceration to the masseter muscle, with exposed mandible and obvious deformity. A significant pulsatile hemorrhage was evident. The patient’s airway was patent, lungs clear, Glasgow Coma Scale 15, and extremities atraumatic.

ED management

Rapid sequence intubation, including the concomitant administration of midazolam, propofol, and succinylcholine, was undertaken, massive transfusion protocol initiated, tranexamic acid (TXA) administered, and the patient prepped for immediate surgery.

Surgical management

**Figure 2:**
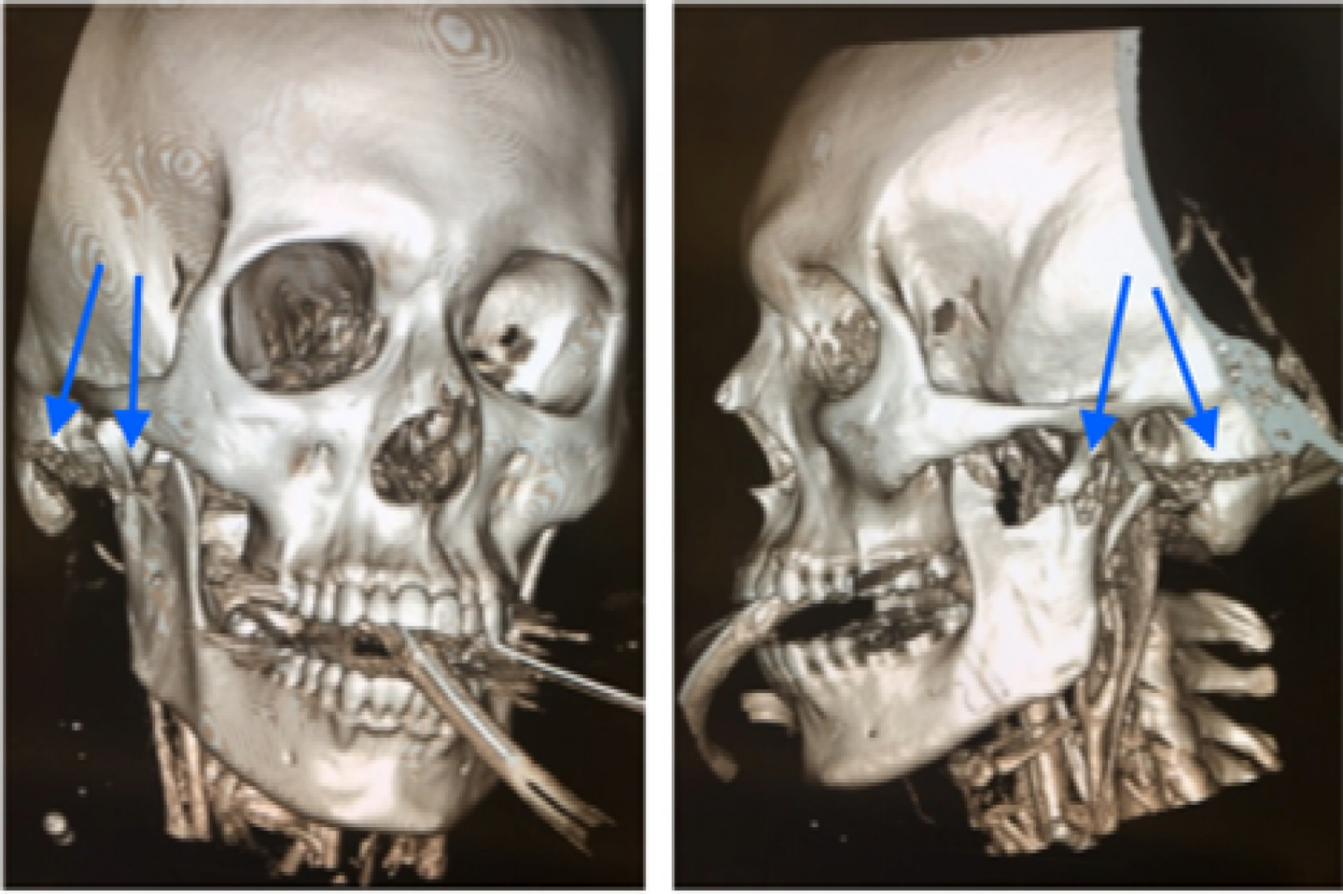
2a and 2b demonstrate a non-displaced fracture of the right mastoid and mandibular condyle

The patient was taken to the operating room by the trauma and oral maxillofacial surgery (OMFS) teams for hemorrhage control, surgical debridement, and wound closure. Following the initial operation, the patient underwent a computed tomography (CT) scan of the maxillofacial structures, which confirmed a non-displaced fracture of the right mastoid and mandibular condyle (Figures 2a-2b). Four days after the initial insult, the patient underwent a right mastoidectomy and facial repair. The OMFS team elected for conservative therapy for the condyle fracture. On discharge, the OMFS team reported a physical exam consistent with House-Brachmann VI.

Outcome

**Figure 3:**
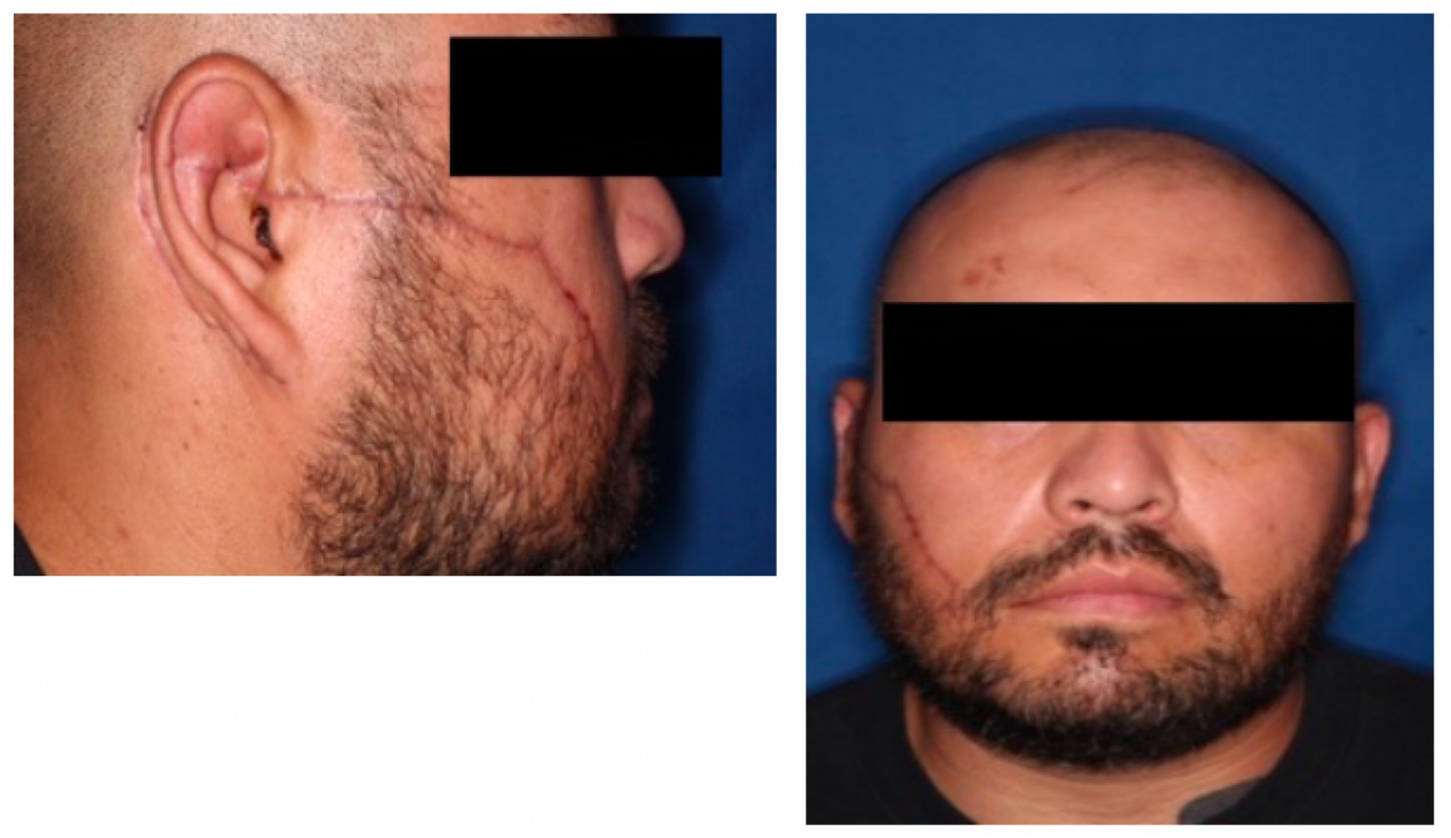
Three-month follow-up demonstrating a well-healed facial wound without signs of infection

The patient was referred to an outpatient neuro-otologist with an immediate follow-up exam demonstrating paralysis consistent with House-Brachmann V. Three months later, the patient’s facial wound had healed well with no signs of infection (Figures 3a-3b). He reported an improved ability to raise his right eyebrow and close his right eyelid as well as difficulty hearing on the affected side. He further reported some difficulty chewing initially but tolerated a liquid diet for eight weeks before slowly progressing to a soft diet. The patient continued to have partial right facial paralysis on examination.

## Discussion

It is important to review both the nature of injury in this case as well as the complex regional anatomy associated with its management. Maxillofacial trauma caused by high velocity or high energy is not common in civilian life, though is well documented in military literature [1,5]. The largest series comes from a review of civilian trainees entering their two years of mandatory military service in Iran. In their study, 20.4% of all injuries in trainees were classified as maxillofacial (903 of 4419) with just 14% of those related to “military causes,” including bullets, shrapnel, and explosions [4].

The safety of the firearm operator and bystanders is dependent on the choice of projectile used, target material, and distance to the target. Manufacturers and gun experts recommend the use of lead-core bullets, including copper-jacketed bullets, which pulverize on impact. These are referred to as frangible bullets, which may also be lead-free [6]. Steel core bullets, sometimes referred to as armor-piercing, are not recommended, as there is a significantly increased risk of the bullet penetrating the target or ricocheting. Of note, frangible bullets have been shown to break during the reloading cycle of semi-automatic weapons; shooting practice with semi-automatic weapons, therefore, poses its own unique risks [7].

The target material must be resistant to deformation from the bullet as well. The hardness of steel is commonly measured on the Brinell scale; steel used in the manufacturing of targets should be greater than 500 on the Brinell scale with a thickness of at least 0.25 inches. Steel any softer than this, regardless of its thickness, is subject to dents and deformation, which creates an increase of bullet ricochet or shrapnel. Of note, manufacturers report that steel any harder than 540 on the Brinell scale is too brittle for use as a shooting target and poses an increased risk of shrapnel-related injury [8]. Finally, the operator must consider his or her distance from the target when shooting at steel targets. Manufacturers vary on their recommended minimum distance depending on: the caliber of the weapon, feet per second of the projectile, and angle of the target [8]. This report is not meant to be a comprehensive review of gun safety, and we recommend operators consult their weapon and target manufacturers for individualized instruction.

Important anatomical structures damaged in our patient’s shrapnel-injury included: mandible, parotid gland, facial nerve, and maxillary artery, including its branches. This review focuses on the maxillary artery and associated branches, including the middle meningeal artery, inferior alveolar artery, deep temporal arteries, buccal artery, palatine arteries, and infraorbital artery [9].

The external carotid artery has two terminal branches: the maxillary artery and the superficial temporal artery. The maxillary artery is the larger of these terminal branches and is further divided into three distinct portions. The first portion, also called the bony portion, includes the deep auricular artery, anterior tympanic artery, middle meningeal artery, and inferior alveolar artery. The second portion, also called the muscular portion, includes the masseteric artery, pterygoid branches, deep temporal arteries, and buccal artery. The third portion, also called the pterygomaxillary portion, includes the palatine arteries (sphenopalatine and descending palatine arteries), infraorbital artery, posterior superior alveolar artery, and artery of the pterygoid canal [10].

Embryologically, the common carotid and internal carotid arteries are derived from the third aortic arch. The external carotid artery and maxillary artery are both derived from the first aortic arch; there are no other remnants of the first aortic arch [11-13]. Given the complexity of the maxillofacial anatomy and embryology, multiple variants have been documented. Variants are primarily noted by clinically insignificant differences in the course of the maxillary artery relative to the lateral pterygoid muscle and the origin of the various branches. Most commonly, the typical branches of the maxillary artery are noted to come directly from the external carotid artery rather than the maxillary artery [13-14].

Clinically, any of the branches of the external carotid artery and even the external carotid artery itself may be ligated without apparent significant ischemic consequences. In fact, for patients with uncontrollable epistaxis, ligation or embolization of the maxillary artery or even the main external carotid artery may be indicated [15-16]. In both civilian and military settings, vascular disruption and hemorrhage are the leading causes of morbidity and mortality. The primary management of vascular trauma requires proximal and distal control with digital pressure, vessel loops, or vascular clamps [17]. Given the complex maxillofacial regional bony and vascular anatomy relationships, such control techniques are challenging in the trauma setting prior to operative exploration. This case further highlights the potential survival benefits demonstrated in the use of TXA in civilian vascular trauma [18-19].

## Conclusions

This case highlights the importance of gun owners matching appropriate ballistics and target selection to avoid a life-threatening injury to both the operator and bystanders. A ballistic-related maxillofacial injury from trauma is uncommon in the civilian population but requires a multidisciplinary team ready to manage both the acute injury and the complex maxillofacial injury. The potential consequences of injury to the maxillary artery and its associated branches are reviewed.
